# Proteomic Methods of Detection and Quantification of Protein Toxins

**DOI:** 10.3390/toxins10030099

**Published:** 2018-02-28

**Authors:** Miloslava Duracova, Jana Klimentova, Alena Fucikova, Jiri Dresler

**Affiliations:** 1 Faculty of Military Health Sciences, University of Defense in Brno, Třebešská 1575, CZ-500 01 Hradec Králové, Czech Republic; jana.klimentova@unob.cz (J.K.); alena.myslivcovafucikova@unob.cz (A.F.); 2 Military Health Institute, Military Medical Agency, Tychonova 1, CZ-160 00 Prague 6, Czech Republic; jiri.dresler@gmail.com

**Keywords:** protein toxins, analytical methods, proteomic, bio-terrorism

## Abstract

Biological toxins are a heterogeneous group of compounds that share commonalities with biological and chemical agents. Among them, protein toxins represent a considerable, diverse set. They cover a broad range of molecular weights from less than 1000 Da to more than 150 kDa. This review aims to compare conventional detection methods of protein toxins such as in vitro bioassays with proteomic methods, including immunoassays and mass spectrometry-based techniques and their combination. Special emphasis is given to toxins falling into a group of selected agents, according to the Centers for Disease Control and Prevention, such as *Staphylococcal enterotoxins*, *Bacillus anthracis* toxins, *Clostridium botulinum* toxins, *Clostridium perfringens* epsilon toxin, ricin from *Ricinus communis*, Abrin from *Abrus precatorius* or control of trade in dual-use items in the European Union, including lesser known protein toxins such as Viscumin from *Viscum album*. The analysis of protein toxins and monitoring for biological threats, i.e., the deliberate spread of infectious microorganisms or toxins through water, food, or the air, requires rapid and reliable methods for the early identification of these agents.

## 1. Introduction

Bacterial and plant protein toxins are among the most powerful poisons known. Protein toxins, especially those of bacterial origin, have harmful effects and often are considered as potential agents used for bio-terrorism and warfare [1]. Therefore, they are relevant in the health and food sector and in the security area. The threat posed by bioterrorism is still a serious concern, as the consequences of a large-scale biological attack would be devastating, causing significant social and economic problems, while being potentially available at relatively modest cost without the need for specific sophisticated technology [2]. According to their above-mentioned properties, biological toxins and their organisms of origin are classified as category A, B, or C priority pathogens depending on how easily they can be spread and the severity of illness they cause [3]. Similar lists exist for control of trade in dual-use items in the European Union [4].

Protein toxins are generally highly toxic to eukaryotic cells and operate via various mechanisms. Many bind to specific receptors on the membrane, most often glycoproteins or gangliosides, and penetrate the cell to achieve their intracellular target [5]. Likely because of this, many protein toxins require two or more subunits to achieve full biological activity, with one subunit binding to the receptor and the second having enzymatic activity (e.g., plant toxins: Ricin from *Ricinus communis*, abrin from *Abrus precatorius*; and bacterial toxins: iota toxin from *Clostridium perfringens*, Shiga toxin produced by *Shigella dysenteriae*, Shiga-like toxins produced by *Escherichia coli*, diphtheria toxin, Pseudomonas exotoxin A, and cholera toxin) [6]. Pore-forming toxins are the largest class of bacterial toxins (e.g., epsilon toxin from *Clostridium perfringens*, toxins from *Bacillus anthracis*) and belong to an ancient protein family also found in non-pathogenic bacteria [7].

Biological threats are difficult to predict and require specific preventive precautions. The response to bioterrorism attacks requires rapid methods that can quickly detect the biological agents used, enabling effective treatment and decontamination. As these agents are effective in very low quantities, detection methods must exhibit both a high degree of sensitivity and a high degree of selectivity to discriminate them from other biological and non-biological material (interference and contamination). One major complication associated with their detection is the complexity and diversity of the samples to be analyzed. In some instances, these agents can be violently delivered by contaminated matrices such as water, food and aerosol or distributed through the postal system (e.g., the anthrax incident of 2001 in the USA, in which ricin was delivered to U.S. government offices in 2004 and, more recently, to the U.S, president and other officials on 16 April 2013) [8]. To date, protein toxins have been identified using various conventional methods involving bioassays or analytical methods, including molecular biology techniques, such as nucleic acid-based assays, i.e., polymerase chain reaction (PCR), and/or immunological techniques, such as enzyme-linked immunosorbent assays (ELISA) or western blotting, for both protein toxins and their producers [9]. These methods are valuable for rapid preliminary screening but may have analytical limitations. Although immunoassays are very sensitive methods compared to mass spectrometry, they may suffer from a lack of specificity and therefore pose a risk of false-positives due to cross-reactions with similar molecules. However, PCR assays are rapid, sensitive and specific but cannot be used for classification of unknown samples or in cases where an isolated toxin is to be detected in the absence of its producer. When there is a higher demand for rapid analysis and a tendency to reach the lowest possible detection limit, these methods are not amenable to absolute protein identification and quantification. Unequivocal detection of potential sources of contamination and the protein toxin itself can be achieved using mass spectrometry (MS). MS is an analytical technique that can provide fast, sensitive and specific analysis in a single technique. MS-based analyses have become a powerful tool in relative/absolute and targeted/untargeted proteomics, also thanks to the continuous development of MS technologies (high resolution, accurate mass HR/AM instruments, hybrid configurations). They are suitable for various analytes, ranging from small organic molecules to large biomolecules and including bioterrorist agents [10]. In addition, MS is a preferred technique according to the Organisation for the Prohibition of Chemical Weapons [11].

## 2. Methods of Detection of Protein Toxins

Early methods for the detection of protein toxins are based on in vivo (e.g., mouse bioassay) or in vitro techniques (e.g., tissue culture). Later, immunological tests were developed together with advanced mass spectrometric (MS) detection, which can give results much faster and are simpler to perform than bioassays. Thus, proteomic methods have brought new views and approaches for the detection of protein-based toxins. These methods involve techniques based on antigen-antibody interactions and sophisticated mass spectrometric detection. Molecular biology methods, such as PCR and its modifications, play an important role in the detection of toxins via indirect methods (detection of DNA as a potential source of contamination). PCR brings more sophisticated approaches (compared to conventional techniques), is much faster and can be applied to detect nucleic acids in most types of environmental matrices [12]. However, interference originating from target-cell lysis—which is necessary for nucleic acid extraction, e.g., nucleic acid degradation and/or direct inhibition of PCR—causes false negative PCR results. Despite the benefits that indirect molecular biology methods provide, in cases of suspected protein toxin contamination in the absence of the producing bacterium, confirmation by direct detection of the toxin is necessary. For this review, PCR-based detection methods are referred to only marginally in chapters covering particular toxins.

### 2.1. Biological Assays

Bioassays and related methods are still the test of choice for the detection of many toxins. These assays require purification of the toxin prior to testing. To perform bioassays, it is necessary to use live animals or plants (in vivo) or tissue cultures and cells (in vitro) to determine the biological activity of toxins. Many bioassay formats have been described, including whole animal tests (e.g., the mouse lethality test, monkey and kitten emesis tests, and rabbit and guinea-pig skin tests), part animal tests (e.g., ileal loop tests) and cell culture systems (e.g., Chinese hamster ovary cells) [13]. Despite enormous progress in the development of alternative in vitro methods, for botulinum neurotoxin (BoNT) detection [14,15], the mouse lethality assay remains the only accepted standard test to confirm active BoNT [16]. The mouse lethality assay has been used for the analysis of complex sample matrices ranging from bacterial cultures to serum, fecal, gastric, or wound samples, but examples of matrix interference with the assay are known, particularly when other substances or toxins present in the sample cause lethality, and it has some other substantial drawbacks [17,18]. Depending on the nature of the toxins to be detected, detection takes from days to months. This factor is limiting in situations when rapid detection and identification is required to protect populations from hazardous biological agents. Nevertheless, the assay is generally considered highly sensitive and specific. The other significant benefit is that it detects functionally active toxin, unlike the majority of immunological methods, which do not provide information about toxin activity.

### 2.2. Proteomic Methods

From a methodological point of view, proteomic methods can be divided into two main categories: methods using antibodies (immunoassays) and advanced proteomic methods based on mass spectrometry.

#### 2.2.1. Immunological Assays

Since their establishment in the late 1960s, immunological assays have become a powerful tool for the detection of proteins. Immunological assays are obviously simpler than biological assays and are therefore widely used [19]. Traditionally, the detection of proteins has been achieved using the enzyme-linked immune sorbent assay (ELISA) [20,21,22] and radioimmunoassay (RIA) [23,24]. Many modified ELISA methods exist [25]. The most expensive and demanding step is to find and purchase an appropriate antibody. However, recent progress in antibody engineering will further support and facilitate the integration of antibodies or antibody fragments into new assay formats [26]. In RIA, the radioactive label is coupled to the antigen that reacts with the specific antibody. The amount of antigen combined with the antibody is then determined using a radioactivity counter. Although the RIA technique is extremely sensitive and specific, the requirement for specialized equipment makes it one of the most expensive methods. The use of RIA for the detection of protein toxins is presented in refs. [27,28,29,30]. Western blotting is another fundamental technique used for the detection and quantification of proteins in complex biological mixtures [31]. Various toxins have been identified according to the western blot results [32,33,34].

Although immunoassay-based methods are sensitive and widely used to measure protein toxins in various matrices, there is a need for methods that can directly confirm the molecular identity of a toxin in situations where immunoassay tests produce positive results. The problem of possible false positivity increases significantly with matrix complexity and requires a confirmatory method. Because immunoassay-based methods require high-quality antibodies due to the high sequence and structural similarity among protein toxins, very few highly specific antibodies are available.

#### 2.2.2. Mass Spectrometry-Based Methods

**Figure 1:**
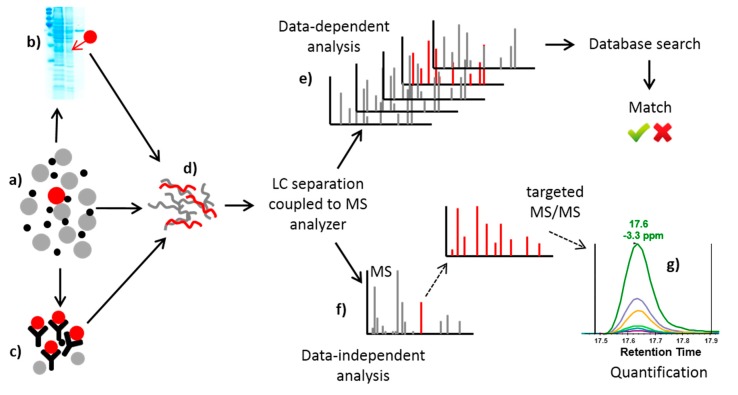
Summary of liquid chromatography–mass spectrometry (LC-MS)-based methods in protein-based toxins analysis: (**a**) protein of interest (red) in complex matrix of other proteins and small molecules; (**b**) SDS-PAGE separation; (**c**) immunoaffinity purification; (**d**) enzymatic digest; (**e**) data-dependent analysis: LC-MS/MS analysis of all peptides in the mixture, all measured MS/MS spectra are searched against protein database, and in the case of a spectral match, the presence of the agent is confirmed, regardless of its quantity. (**f**) In a targeted (data independent) MS/MS experiment, peptides of interest are fragmented repeatedly during their elution times, and (**g**) extracted ion chromatograms of selected fragment ions are then used for protein quantification (exported from Skyline, unpublished results).

A wide range of MS-based methods for protein toxins detection is available [8,10,35]. These methods are highly advantageous in complex matrices, where they profit from combination with various separation techniques, such as one-dimensional and two-dimensional polyacrylamide gel electrophoresis and gel-free approaches based on liquid chromatography. The above-mentioned antibody-based techniques have great advantages when used to concentrate the analyte of interest and reduce the complexity of the matrix prior to MS analysis. In combination with MS detection techniques, they represent a powerful tool in the detection and quantification of protein toxins. See Figure 1 for a summary of MS and separation methods used in protein toxin analysis.

The MS methods comprise two different approaches. In data-dependent experiments, all peptides present in the mixture are fragmented, and their MS/MS spectra are searched against a relevant protein database. Nevertheless, this type of experiment does not give any information about the quantity, and the criteria for match relevance (number of peptides and their scores) are the researcher’s choice. Thus, this type of experiment often serves as a preliminary test for the presence of biological agents in the case of unknown samples, and the results must be further supported by targeted analysis. However, targeted (data independent) experiments are relevant for quantitative and confirmative cases. Here, the method targets previously selected peptides of interest and only scans and fragments these peptides. Targeted (data independent) MS/MS experiments are based on two principles, each taking advantage of a different type of mass spectrometer. First, selected reaction monitoring (SRM) [36] is performed on triple quadrupole (QqQ) mass spectrometers. In this experiment, the first quadrupole serves as a mass filter that targets a particular peptide and sends it further to the second quadrupole, which operates as a collision cell to fragment the peptide. The specific product ion of the fragmentation reaction is then filtered by the third quadrupole. The specific pair of *m*/*z* values associated with the precursor peptide and its selected fragment ion is referred to as a “transition” and is usually reported as the parent *m*/*z* > fragment *m*/*z*. Unlike the classical data-dependent MS experiment, full mass spectra are not recorded in a SRM analysis. Instead, the detector acts as a counting device for the chosen specific ions. Some of their fragments return an intensity value over time [36]. The principle of the second method, parallel reaction monitoring (PRM), is similar to that of SRM but is performed on hybrid quadrupole-Orbitrap mass spectrometers. The targeted precursor ion is filtered and fragmented as described above, but the whole MS/MS spectrum is recorded using the Orbitrap mass analyzer. This allows the quantification of tens to hundreds (and thousands in wide-screen analyses) of targeted proteins in the same run [37]. SRM- and PRM-based methods rely heavily on instrumentation as well as software capable of targeted proteomics method creation and quantitative data analysis. Open-source Skyline software (Version 4.1, the MacCoss Group at the University of Washington, Seattle, WA, USA, released on 11 January 2018) was explicitly developed to aid in this targeted assay development [38]. Quantification in SRM and PRM experiments is performed by extracting the product ion chromatograms and calculating the area under the curve. Absolute quantification of protein (also called AQUA) was described in the early 1980s [39]. The AQUA approach is based on addition of a known quantity of stable isotope-labeled standard peptides. The signal intensity of the target peptide in the experimental sample is compared to that of the heavy peptide and is then back-calculated to the initial concentration of the standard using a pre-determined standard curve to yield the absolute quantification of the target peptide. The development of a quantification method is completed by its validation, which comprises assessment of the detection and quantification limits [40,41]. These limits are further decisive in forensic cases when the presence of a particular toxin is to be reported. Absolute quantification for several protein toxins in various complex matrices has been described [42,43,44]. There is another important group of detection methods that focuses on protein toxins with intrinsic enzymatic activity (such as *Bacillus anthracis* and *Clostridium botulinum* toxins) [45]. These methods are based on the combination of biochemical tests with indirect MS detection of the enzymatic reaction products. Their use was first described in 2005 in botulinum neurotoxins [46] and are discussed below.

Compared to the methods using an antibody-antigen interaction (e.g., Western blotting and ELISA), SRM/PRM have several advantages. First is the quality of the assay, as quantification by western blotting is based on a single reagent (antibody), which may be poorly characterized. In contrast, SRM/PRM assays depend on isotopically labeled reference peptides, the quality of which can be easily verified by a fragment ion spectrum. There is also an economic advantage. Given the primary investment in a mass spectrometer and operating costs for the facility, it is still notably cheaper to develop SRM/PRM assays than to screen antibodies for each targeted protein. Finally, performance characteristics, such as limit of detection, linear dynamic range, ability to multiplex, and reproducibility, are also superior in MS-based methods.

## 3. Detection of Selected Protein Toxins

### 3.1. Staphylococcal Toxins

*Staphylococcus* is a genus of Gram-positive bacteria that includes approximately 40 species. Most are harmless and reside on the skin and mucous membranes of humans and other organisms. Nevertheless, some are important as human pathogens. Due to the diversity of this genus, *Staphylococci* cause a great variety of infections, including skin infections, pneumonia, food poisoning, toxic shock syndrome, and blood poisoning (bacteremia) [47]. The most important species from a toxicological point of view is *Staphylococcus aureus*.

*Staphylococcus aureus* is a Gram-positive, round, facultative anaerobe bacterium frequently found in the nose and the respiratory tract and on the skin. *S. aureus* is not always pathogenic but is a common cause of skin infections such as abscesses, respiratory disease and food poisoning. Pathogenic strains induce infections by producing various virulence factors such as potent protein toxins and expression of a cell-surface protein that binds and inactivates antibodies [48]. For the purpose of this review, the detection and identification of *S. aureus* enterotoxins (SEs) will be summarized.

Staphylococcal enterotoxins are secreted proteins of approximately 30 kDa that interact with intestinal mucosa and cause emesis and diarrhea. Currently, 23 enterotoxins have been identified as distinct serological individuals [49]. The most common SEs are *S. aureus* enterotoxin A (SEA) and B (SEB). SEA plays an important role in food poisoning caused by *Staphylococcus* [50]. SEB is one of the most potent bacterial superantigens, and their toxic effects are based on the activation of cytokine release, ultimately causing cell death by apoptosis. Currently, no treatment or vaccine is available [51]. SEB had been considered and produced as an offensive biologic warfare agent and is identified as a restricted agent by the CDC (Centers for Disease Control) [52].

• Detection of *Staphylococcus aureus* enterotoxins

The first methods for the detection of SEs were conventional methods such as animal [53,54] and serological tests [55]. The development of molecular biology methods such as PCR brought more sophisticated and faster approaches to detect SEs [12]. Furthermore, there are well-established and sensitive immunoaffinity-based methods available for SEs in various matrices, many of which have detection limits in the range of 1–10 ng/g. Three are three main types of sensors for SE identification using these methods: optical, electrochemical and mass detection techniques. Furthermore, optical detection methods based on colorimetric [56,57,58,59,60,61,62], fluorescence [63,64,65] and chemiluminescence [66] principles as well as highly sophisticated methods, such as electrochemiluminescence [67] and the surface plasmon resonance (SPR) [68] immunoassay, have been developed. ELISA is a fundamental and widely used colorimetric method and is generally the most common method of SE detection. Electrochemical immunoassays [69] present the newest method for simple, sensitive, portable, cheap, and reproducible SEs detection and has outstanding compatibility with the latest technologies. Nevertheless, commercial kits are only available for the detection of five enterotoxins (SEA to SEE) and suffer from serious limitations in terms of sensitivity, specificity and suitability for complex food matrix analysis. A list of methods of SE detection are summarized in Table 1.

Mass spectrometry-based methods, on the other hand, provide abundant specific information. MS analysis of intact SEB was reported and published in the early 1990s [78]. Rapid isolation and identification of staphylococcal exoproteins by LC/MS—followed by the determination of N-terminal amino-acid sequences of separated peaks—was developed [79]. Applications involving the detection of SEs in food matrices have become widely used. Biomolecular interaction analysis mass spectrometry was applied to detect bacterial toxins in food samples [80]. The SEB concentration in milk and mushrooms was determined using a combination of antibody extraction and matrix-assisted laser desorption/ionization mass spectrometry (MALDI-MS). Quantitative MS analysis of SEB in food matrices using labeled peptides and a label-free approach was performed [81]. The objective of this study was to demonstrate that a proteomics-based strategy can be effectively used to detect and quantitate SEB in food matrices within the accepted criteria for bioassays. Another protein standard absolute quantification (PSAQ) strategy represents the ideal alternative methodology, allows the evaluation of the digestive pathogenicity of poorly characterized SEs and helps investigate staphylococcal food poisoning outbreaks that cannot be solved with existing immunological tools [42]. Reference [82] shows how proteomics-based methods can be effectively used to detect, confirm and quantitate SEB in food matrices. Amine-modifying labeling reagents were used to quantitate the protein in complex food matrices using differential isotopic tags, reference internal standards and LC-MS/MS analysis. In this study, the authors show an alternative labeling strategy using acetylation with acetic anhydride (Ac2O/2H6-Ac2O) that is reasonably priced, reliable and provides adequate information about SEB in food matrices. In addition, this method does not require SEB to be manipulated during routine analysis and therefore represents a significant advantage for laboratories controlled by regulatory agencies. Another report presents a label- and antibody-free alternative method based on a bottom-up proteomics approach for the targeted measurement of SEA and SEB in milk and shrimp with UPLC-ESI-MS/MS. This analysis of toxins uses proteotypic amino-acid sequences within the toxin. The use of corresponding ^13^C-labeled internal standard sequences and tandem mass spectrometry provides high specificity during simultaneous identification and aids the quantification of SEA and SEB toxins in highly complex food extracts at low ppb levels. As the method can be applied without the need for specific reagents or antibodies, it may be extended to other food matrices and enterotoxins for which antibodies have not been developed [83]. The most up to date methods for the detection and identification of SEs in food matrices are shown in refs. [84,85]. In the proteomics-based bottom-up approach [84], the LC technique combined with MS/MS in multiple reaction monitoring (MRM) mode was used to detect and quantify two types of SE (A and B) spiked into a milk and buffer solution. In the proteomic assay developed for simultaneous detection of several CBRN-relevant toxins in food, the PSAQ approach for highly specific detection and absolute quantification strategy was used [85]. Here, CBRN stands for chemical, biological, radiological and nuclear protection. The assay was based on an antibody-free sample preparation followed by bottom-up LC-MS/MS analysis operated in targeted mode. An overview of the newest identification methods of SEs by mass spectrometry is shown in Table 2.

### 3.2. Bacillus anthracis Toxins

*B. anthracis* is a Gram-positive, pore-forming, rod-shaped bacterium and the only obligate pathogen from the genus *Bacillus.* It is one of only a few bacteria known to synthesize a protein capsule (poly-d-gamma-glutamic acid). It forms a calmodulin-dependent adenylate cyclase exotoxin known as anthrax edema factor, along with anthrax lethal factor. Genotypic and phenotypic similarity to *B. cereus* and *B. thuringiensis* has been shown, and all form oval endospores. *B. anthracis* endospores are highly resilient, surviving extremes of temperature, low-nutrient environments, and harsh chemical treatment over decades or even centuries. Because of these features, *B. anthracis* endospores are particularly well-suited for use (in powdered and aerosol form) as a biological weapon [86]. The U.S. CDC classifies *B. anthracis* as a category A biological agent [87]. Thus, there are many reasons to study the mechanism of action and methods for detection of the microorganism and its protein toxins. The virulence of *B. anthracis* species is primarily caused by different components: The poly-d-glutamic acid capsule and the anthrax toxin. The genes for capsule biosynthesis and anthrax toxin are coded by double-stranded DNA situated on plasmids, called pX01 and pX02 [35]. The anthrax toxin is composed of three distinct proteins: protective antigen (PA), lethal factor (LF), and edema factor (EF). Lethal (LeTx) and edema toxin (EdTx) each contain 2 protein types. LeTx is composed of oligomers of PA and LF, and EdTx is made up of PA and EF. These proteins must act together [88]. PA is an 83 kDa protein, and it provides the toxin entrance into the cell. After administration of toxin to the body, PA binds to receptors on the cell surfaces (most often through macrophages) and is cleaved by protease to a 63 kDa fragment, which is attached to the receptor. PA63 forms an oligomer at the cell surface that binds three molecules of LF and EF. EF is a calmodulin-dependent adenylate cyclase that converts ATP to cyclic AMP (cAMP). Decreasing levels of ATP leads to a decrease in chloride ions and water from the affected cells, which leads to the massive edema observed in cutaneous cases of anthrax. EF also inhibits the phagocytic and oxidative burst activity of neutrophils, which increases the vulnerability of the host to spread the infection. LF is a zinc-dependent metalloprotease that cleaves several members of the mitogen-activated protein kinase (MAPK) and thus inhibits the signal transduction pathway. At this point, increasing levels of the cytokines TNF-α and IL-1β in macrophages have been reported. LF activates the oxidative burst pathway, leading to the production of reactive oxygen intermediates [89]. Both toxins act in concert to cause immune deregulation, endothelial dysfunction, advanced septicemia, hemorrhage, and shock, which often leads to death [35].

• Detection of *Bacillus anthracis* toxins

Detection of *B. anthracis* microorganism and its toxins have been described in many studies. Some genetic similarities to other members of the genus *Bacillus* (*B. cereus*, *B. thuringiensis* and *B. mycoides*) make the detection of *B. anthracis* and its released toxins challenging [90]. Conventional microbiological methods based on morphological examination of the growth culture have long been a gold standard for its identification [35]. Other methods based on the biochemical properties of the organism have been described and usually act as confirmation methods on isolated colonies that are already suspected of being *B. anthracis* based on their morphological characteristics. Most of the assays are based on detection of the whole organism, bacterial antigens, and/or the nucleic acid. A summary of relevant detection methods of *B. anthracis* is shown in Table 3.

Enzyme immunoassays detect *B. anthracis* toxin proteins such as PA, EF, LF or the poly-d-glutamic acid capsule [112,113]. Many immunoassay formats are currently commercially available for a wide variety of detection needs and are outlined in Table 2. The mechanism of action of *B. anthracis* toxin has been described before, and it is clear that its enzymatic activity can be used to benefit detection. The zinc metalloprotease activity of the anthrax lethal factor was studied for these purposes. Notably, the detection of toxin-specific reaction products may often be easier than the detection of the toxin itself [35,45]. One technique termed Endopep-MS was first mentioned in 2005 [111]. In this study, the toxin activity quantification by MS is based on three levels of measurement. First, the toxin is purified/enriched using monoclonal antibodies (mAbs) covalently bound to magnetic beads that are specific for the toxin. The second step includes toxin-specific enzyme activity directed against a specific substrate, and the last step is MS detection of toxin-generated reaction products [35,45]. Total LeTx and EdTx are captured from the clinical sample using mABs. The captured LF is then added to a reaction buffer containing an optimized peptide substrate with an amino acid sequence, similar to the portion of MAPK cleaved by LF, to give two products with a specific mass. The remaining substrate and two LF-specific products are detected by MALDI-TOF. The EF is caught by mAbs and incubated with ATP and the adenylyl cyclase cofactor calmodulin. The ATP and adenylyl cyclase reaction product cAMP are detected by LC-ESI-MS/MS. Furthermore, the use of isotopically labeled cleavage product allows the use of the method for quantification of LF [111]. The main advantage of monitoring anthrax toxin by MS is early detection of LF in less than 12 h. The LF can be detected before positive bacteremia status, before detection by PCR, and before detection by antigen capture immunoassay [35]. Clinical applications of Endopep-MS using MALDI-TOF-MS were published [114,115].

### 3.3. *Clostridium* Toxins

*Clostridium* is a genus of Gram-positive bacteria that contains significant human pathogens, including the causative agent of botulism, and is an important cause of severe necrotizing disease in the large intestine. The *Clostridium* genus comprises approximately a hundred species. The most interesting species related to human and animal diseases are: *C. botulinum*, *C. difficile*, *C. perfringens* and *C. tetani*.

Members of the *C. botulinum* strain produce botulinum neurotoxins (BoNT). The diverse group of *C. botulinum* strains is sorted into four distinct groups according to their ability to produce toxins. Human pathogenic neurotoxins of types A, B, E, and F are produced by the *C. botulinum* groups I and II, *C. butyricum* and *C. baratii* [14]. BoNT are the most lethal bacterial toxins known to mankind and are produced by both natural and synthetic routes, with a lethal dose of approximately 2 ng/kg [116]. BoNT are categorized into seven serotypes from A to G based on their response to antisera. All seven serotypes are synthetized by the bacterium as single-chain polypeptides. The different serotypes are approximately 40% similar at the amino acid level [45]. BoNT are composed of an enzymatically active light chain (LC, 50 kDa) connected by a disulfide bond with a receptor binding heavy chain (HC, 100 kDa). The HC is comprised of two regions: the amino terminal 50 kDa domain with a translocation function and a 50 kDa carboxyl terminal domain. LC domains with zinc-dependent endoprotease activity are responsible for the inhibition of neurotransmitter release. There are three membrane proteins on the presynaptic cells (secretory vesicle) that serve as a target for this endoprotease [35]. The BoNT serotypes have different functional properties. BoNT A, C and E hydrolyze synaptosomal associated protein 25 (SNAP-25), and BoNT B, D, F and G hydrolyze isoforms of synaptobrevin, also known as vesicle associated membrane protein (VAMP-2). BoNT C is unique within the group, as it hydrolyzes a second substrate, syntaxin, in the vesicle docking complex [117,118]. Due to its extreme toxicity, speed of the initiation of symptoms and lack of treatment, a sensitive and rapid BoNT detection method is needed to diagnose botulism in suspected cases before paralysis occurs. Methods of BoNT detection have been reviewed in many publications [14,15,16].

• Detection of BoNT

While the mouse lethality assay has remained the standard test for the detection of botulinum neurotoxins, there has been great progress in the development of alternative in vitro tests in recent years. The mouse lethality test is based on intraperitoneal injection of mice with dilutions of BoNT-suspected samples and sequential observation of these mice for symptoms of botulism and ultimately death, which requires a fully functional toxin capable of binding and entering neurons before cleaving its SNARE (an acronym derived from “SNAP (Soluble NSF Attachment Protein) receptor substrate. It is necessary to find both the maximum sample dilution that kills mice and the minimum dilution that does not kill to estimate the quantity of BoNT in the sample. If the dilution that does not kill is not found and all injected animals die, the sample must be diluted further and the procedure repeated [119]. The quantity of toxin in the sample is then estimated by relating the maximum dilution that kills to the known mouse-lethal dose (MLD50). The toxin serotype is then determined by neutralization of the toxin with serotype-specific antitoxin, which is usually administered prior to toxin injection. Mice are observed for signs of botulism for another 48 h to deduce which specific antitoxin is protective. The mouse bioassay is applicable to all BoNT serotypes, with an LOD of 5 to 10 pg of BoNT/A, but a minimum of four to six days is needed. Variations in the mouse lethality assay have been developed to reduce the number of laboratory mice required [120,121,122,123]. All of these methods can estimate toxin quantity using significantly fewer animals than the mouse lethality assay, but the toxin serotype must be known in advance to correlate symptoms and survival times to dose. If the serotype is unknown, a toxin neutralization assay or alternative assay must be performed.

PCR techniques to identify the presence of *C. botulinum* DNA were originally used to detect the presence of bacterial spores. The method can detect the presence of as few as 10^2^ spores per reaction mixture for serotypes A, E and F and only 10 spores per reaction mixture for BoNT/B. To enhance the sensitivity, Lindström and colleagues developed an enrichment method that could detect as few as 10^−2^ spores/gram of sample for the serotypes A, B and F and 10^−1^ spores/gram of sample for BoNT/E [124]. One critical shortcoming of this method is that enrichment often requires 5 days. Furthermore, the applicability of the assay for the detection of food contamination was diminished by the observation that food matrices could interfere with the sensitivity of the assay. Additionally, if contamination were to occur with the actual toxin and not cells, this traditional PCR method would not be useful. Multiplex technology is conceptually simple for PCR-based assays. Different sets of PCR primers can easily be generated, each highly specific for a gene of interest, allowing for the amplification of multiple targets in one reaction tube. Many multiplex technologies for the detection of BoNT genes have been described [125,126,127,128]. The main disadvantage of the molecular detection of BoNT is the impossibility to detect gene or toxin activity.

Numerous immunological tests have been developed, although many earlier assays, such as radioimmunoassays [129,130], passive hemagglutination [131,132] or the gel diffusion assay [133], have poor sensitivities or specificities. After all, these assays were overcome by more sensitive ELISA. After over four decades of use for BoNT detection [134,135], ELISA is the most commonly applied immunological test. Several variations of ELISA protocol were developed to enhance assay sensitivity [136,137,138,139]. ELISA methodologies have been successfully employed in the detection and quantification of purified *botulinum* toxin, in *C. botulinum* cultures that produce the toxin [140], in an extensive variety of food samples [141] (both contaminated food and food artificially spiked with the toxin), and in clinical samples such as serum [142] and feces [143]. Some foods tend to interfere with ELISAs and decrease their sensitivity; therefore, the results should be confirmed by a mouse lethality assay.

The serotype specificity of the ELISA depends on the specificity and cross-reactivity of the antibodies used. All BoNT serotypes are immunogenic and can elicit production of antitoxin antibodies. The seven serotypes differ by up to 70% at the amino acid level, enabling the selection of antibodies with little or no cross-reactivity [144]. Both monoclonal and polyclonal antibodies have been used in ELISA experiments to detect and serotype BoNT, with polyclonal more common due to reduced procurement costs and easier accessibility. Polyclonal antibodies with high specificity against serotypes A, B, E, and F (serotypes generally causing human disease) were employed to identify these BoNT serotypes by amplified-ELISA and ELISA using digoxigenin-labeled antibodies [136] with high sensitivity and no cross-reactivity. Sensitive ELISAs relying on mABs with high serotype specificity have also been developed [145]. Genetic variation within the different serotypes of the toxin may result in decreased affinity for mABs, causing false-negative results [144].

One widely used technology for the detection of peptide bond cleavage is fluorescence resonance energy transfer (FRET) [146]. Multiple variations of fluorophore/quencher modified substrates derived from SNAP-25 have been developed and used for the detection of BoNT/A [147,148].

BoNT has enzymatic activities as well as the previously mentioned *B. anthracis* toxin. BoNT are Zn^2+^-dependent endopeptidases that inhibit neurotransmitter release via the specific cleavage of synaptic SNARE complex proteins. Exploitation of this endopeptidase role of BoNT has led to numerous detection methods. Unlike the immunological in vitro methods described above, which are unable to discriminate between an active and inactive form of the toxin, endopeptidase assays detect the active form only. For example, if food that was heated was positive for the presence of BoNT by immunoassay, it may be negative by the mouse lethality assay and endopeptidase assays, as the toxin may be inactive. In this sense, endopeptidase assays are more similar to the mouse lethality bioassay than immunoassays. The first assay based on the endopeptidases activities of BoNT was developed in the early 1990s. In this assay, synthetic peptides with a specific cleavage site were added to the sample, and cleavage products were detected by ELISA [149]. The method was specific to BoNT/B, showing no cross-reactivity with other clostridia neurotoxins, and had sensitivity from 0.6 to 4.5 ng/mL. A rapid endopeptidase-MS to detect and differentiate active BoNT A, B, E, and F was developed [150]. This method has found utilization in various matrices [151]. In the study, in which all conventional tests were used, *botulinum* toxin was successfully detected in patient serum using the endopeptidase-MS assay, although all conventional tests gave negative results [152].

In recent years, MS analysis has become a promising choice for the characterization and detection of BoNT. A method previously developed for the MS identification of tetanus toxin was first applied to BoNT/A and B [153] as well as BoNT C, D, E, and F to complete the characterization of *botulinum* toxins [154]. In this study [155], nanoLC-MS/MS with peptic sample pretreatment and neurotoxin database identification was used to characterize the protein composition of *botulinum* progenitor toxins and assign *botulinum* progenitor toxins to their proper serotype and strain using currently available sequence information. In contrast to other investigations in this study, crude *botulinum* toxins directly from proteolytic *C. botulinum* strains were used rather than standard materials and demonstrated accurate identification of highly toxic *botulinum* toxins based on peptide sequences. The results demonstrated that the combination of peptic pretreatment and multidimensional nano-LC–MS/MS with an ion trap detector represents substantial progress in *botulinum* toxin detection [116]. A summary of methods relevant to the characterization of BoNT is in Table 4.

In summary, the detection of BoNT at relevant concentrations is challenging because BoNT are extremely lethal, and therefore the test must be correspondingly sensitive. The currently accepted test for functional detection of BoNT is still the standard mouse lethality bioassay, although it is time consuming, costly, impractical for screening large numbers of samples and cannot be used in the field. A fully functional, highly sensitive replacement assay is the mouse phrenic nerve hemidiaphragm assay, which is currently being validated for pharmaceutical products [156,157]. Assays that detect BoNT proteolytic activity employ naturally occurring or synthetic analogs of SNARE proteins combined with methods for detecting the cleaved products such as FRET or MS.

*Clostridium perfringens* is a Gram-positive anaerobic spore-forming bacterium that is widely distributed in nature, especially in environmental matrices such as soil or water and the intestinal tracts of humans and animals. Under natural conditions, this bacterium is responsible for local outbreaks of food poisoning associated with the consumption of contaminated food and is frequently connected with inappropriate storage or treatment of food. The bacterium is also a major cause of gas gangrene. The absence of early treatment leads to the spread of toxins in the body, causing shock, coma and death. *C. perfringens* has been shown to produce 18 toxins: alpha (CPA), beta (CPB), epsilon (ETX), iota (CPI), enterotoxin (CPE), theta/perfringolysin O (PFO), beta-2 (CPB2), TpeL, NetB, NetF, BecA/B, NanI, NanJ, kappa, mu, lambda, clostripain, and delta toxin. *C. perfringens* strains can be divided into five types (A to E) based on the production of four major extracellular toxins (α, β, ε, and ιA) [158]. *C. perfringens* types B and D produce the epsilon toxin, which is considered the third most powerful clostridial toxin. Due to the possibility to disperse the toxin as an aerosol and a lack of methods for the treatment and prevention of poisoning, *C. perfringens* spores and toxins are considered biological warfare agents. See Table 5 for the diversity of *C. perfringens* toxinotypes and associated diseases [159].

Alpha toxin is a 45 kDa necrotizing toxin, which in purified form has been shown to be a zinc-containing phospholipase C enzyme. Its encoding gene (*cpa*) is present in all *C. perfringens* strains. Studies have shown that alpha toxin is the major virulence factor in cases of gas gangrene [160]. Beta toxin (38 kDa) is produced by the B and C types and it is the primary lethal factor in type C. Beta toxins have 28% structural similarity to pore-forming *S. aureus* alpha toxins, and it is expected that beta toxin acts in a similar way [161]. Epsilon toxin (32 kDa) is produced as a single-chain prototoxin by the B and D types. Epsilon toxin is a pore-forming protein that causes potassium and fluid leakage from cells and is considered a potential biological warfare agent in category B [162]. Iota toxin is known as an AB toxin produced by E strain and is composed of two different proteins: Ia (52 kDa), which has enzymatic activity and Ib (98 kDa). Ib binds to receptors on targeted cells and translocates Ia into the cytosol. Ia causes ADP-ribosylation of actin, resulting in cell rounding and death [163]. The *C. perfringens* toxins related to human and animal disease are enterotoxins and beta-2 toxin. Enterotoxin is the major toxin responsible for human food poisoning, and beta-2 toxin is a recently described toxin responsible for porcine necrotic enteritis [164].

• Detection of *Clostridium perfringens* toxins

The bacterium and especially its toxins are usually targets of interest for clinical laboratories, the food industry and biodefense. Immunological [165], molecular biology [166] and MS analysis through application of MALDI-TOF identification [167,168,169,170] are applied in these specialized laboratories. Due to the wide diversity of toxins of *C. perfringens*, many studies on their detection and identification have been published. In general, the two main molecular approaches for detection of *Clostridia* toxins are EIA [171] and PCR [172,173,174]. EIA methods are rapid and sensitive and are typically used to target toxins [175,176]. However, cross reactivity is a common problem leading to a high false positive rate, which can misguide the public health response; additionally, the toxins of *C. perfringens* are unstable and can degrade quickly, which can lead to false negatives if stool or food samples are not analyzed soon after collection or are subjected to improper laboratory conditions. Real-time PCR methods are typically employed to amplify toxin genes. Difficulties with this method can arise, since many of the toxin genes in *Clostridium* spp. reside on extrachromosomal elements (e.g., plasmids or phages) and can be horizontally transferred to other types of bacteria or even within *Clostridium* species. The transfer of genetic material between them could be problematic for methods targeting only one or a few genes from a single species. Those drawbacks could be overcome by characterization of *Clostridium* species utilizing LC-MS for intact proteins [177]. Biodefense laboratories focus on the detection of epsilon toxin as a potential biological warfare agent. The *C. perfringens* epsilon toxin is the third most potent clostridial toxin in nature following BoNT and tetanus toxins [162]. Since introducing non-animal alternatives, the classic mouse assay involving toxin neutralization with *C. perfringens* type-specific antisera has been replaced. ELISA technology for specifically detecting ETX in intestinal contents is sensitive and quantitative, giving excellent agreement with the mouse protection test, and thus it is one of the best ways to confirm poisoning [178,179]. Molecular biology methods can identify the ETX gene if it is present [180]. Detection and quantification of the ETX protein is also possible using a novel MS technique [168,181]. MS avoids cross-reactivity issues that are intrinsic to any antibody-based assay, but both immunoassays and MS do not determine the presence of the biological activity of toxins. For this purpose, a latex agglutination test has been developed and published as a cytotoxicity assay using Madin-Darby canine kidney cells [182]. A summary of methods relevant to the characterization of *C. perfringens* and its toxins is shown in Table 6.

### 3.4. Selected Plant Protein Toxins

Plants cannot move to escape their predators, so they must have other means of protecting themselves from herbivorous animals. Some plants produce highly toxic protein toxins, several of which are considered biological warfare agents by the CDC. Two significant plant protein toxins from a biodefense and toxicology point of view, namely, ricin and abrin, are produced by *Ricinus communis* and *Abrus precatorius*, respectively. Both belong to the family of ribosome-inactivating proteins (RIP). Proteins from the RIP group inactivate the 60S ribosomal subunits of eukaryotic cells by *N*-glycosidic cleavage, which releases a specific adenine base from the sugar-phosphate backbone of 28S rRNA. They are both naturally occurring lectins with a similar dimeric structure consisting of two protein subunits connected by disulfide bridges. The A-chain is responsible for inhibiting protein synthesis, and the B-chain is a galactose-specific lectin responsible for binding of the toxin to the cell membrane, which precedes its endocytosis. Abrin (58 kDa) is soluble in water and naturally occurs in the seeds of the *Abrus precatorius* (rosary pea), plant, which is common to tropical regions. Ricin occurs in the seeds of the castor oil plant *Ricinus communis*. The A-chain and B-chain are of similar molecular weights, approximately 32 kDa and 34 kDa, respectively. In addition to these best-characterized RIP toxins, the class of plant toxins includes lesser known potent chem-bio threat agents, such as viscumin (*Viscum album*), modeccin (*Adenia digitata*), and volkensin (*Adenia volkensii*) [183]. Immunoassays and functional tests are available for the determination of RIP toxins. In functional assays, such as the luciferase luminescence test [184], the enzymatic activity of the A-chain is determined using a cell lysate. Other functional tests assess the enzymatic activity of the A-chain against RNA, using MS to quantify the hydrolysis of adenine in a substrate molecule that is added to the sample [185]. Cell-based cytotoxicity tests that assess the toxin’s full functionality in complex matrices are available [186]. These tests are highly sensitive and can detect toxin activity at concentrations of less than 1 ng/mL. Immunoassays, such as ELISA, using specific antibodies have demonstrated similar sensitivity [187,188].

Ricin from *Ricinus communis*. Ricin is a naturally occurring toxin found in the seeds of the castor plant (*Ricinus communis*), which is globally cultivated and processed in large quantities. Ricin has been used as a biothreat agent in the past and has gained national attention due to its remarkable toxicity. The toxicity associated with ricin has long been established, with over 700 human intoxications reported dating as far back as the late 1800s [189]. A summary of notable accounts is summarized in reference [190], including a recent case report of a fatality due to the ingestion of an herbal product containing a lethal level of castor bean powder [191]. In many other recent examples, antigovernment and terrorist groups were involved in the attempted use of ricin as a bioweapon. Its history as a weapon has led to the categorization of ricin by the CDC as a category B biothreat agent [190].

• Detection of ricin

In the case of an intentional release of ricin into the environment, the discrimination of functionally active and denatured ricin is important, especially with regard to emergency operating schedules, forensic analysis and therapy. This information can only be obtained from functional assays, which can be principally differentiated into assays detecting A-chain activity, the B-chain activity, or both. However, the detection of the activity of the isolated subchains provides no information about the activity of the intact 64 kDa ricin molecule. Therefore, the detection of active ricin requires in vivo [192] or in vitro [185] assays for both subchains. To detect functionally active ricin, two methodologies have been used: monitoring of the ribosome inactivating A-chain-induced inhibition or measuring of the release of adenine or depurinated product from the RTA cleaved substrate; the latter method is more convenient. In functional assays where the hydrolysis product is monitored, ricin is usually captured or enriched from environmental samples using an immunoaffinity technique and then reacted with a substrate [193]. The release of labeled or unlabeled free adenine from a ribosome or a synthetic oligonucleotide substrate can be detected by various detection techniques, such as chemiluminescence [194] and MS [195]. Traditionally, immunological methods have been used to detect ricin [196]. Castor seeds contain ricin but also a second lectin, *Ricinus communis* agglutinin (RCA), which is structurally related to ricin, with over 93% similarity between A-chains and 84% similarity between the B-chains. However, RCA is less toxic [197], which is relevant when choosing an appropriate analytical method. ELISAs can be very sensitive but inclined to non-specific reactions and may give positive responses for RCA, even for denatured ricin lacking toxic activity [198]. In vitro toxicity assays and molecular methods such as PCR provide information on the type of toxic action but do not provide absolute evidence of the substance present in the sample. In some cases of ingestion of ricinus material, the low molecular weight alkaloid ricinin has been used as a marker, as it is easier to detect this compound than the protein ricin [199]. The first MS-based activity test of ricin was presented in 2000 by Fabris, who detected ricin-induced depurination of RNA by direct infusion of ESI. Before this time, MS-based analysis had not produced any direct information about the activity of the toxin. Many other publications have followed [185,195,200,201,202,203]. In reference [193], the sensitivity for the qualitative and quantitative analysis of active ricin by MALDI-TOF MS has been improved. In reference [190], ricin detection methods that can and cannot detect biological activity are reported. Many detection methods provide robust, sensitive, and quantitative detection of ricin, and detection assays that can distinguish between biologically active and inactive ricin are essential for evaluating the lethality of a bioterrorism threat and for monitoring site decontamination procedures. However, many of the biological assays discussed here that can detect toxicity and A-chain activity have limitations in selectivity and cannot distinguish ricin from other harmful toxins. For these reasons, it is necessary to utilize an integrated approach in the development of an ideal ricin detection method. The optimal assay design would have a rapid and efficient enrichment step, an A-chain activity checkpoint, and a selectivity step that can distinguish ricin from other bioactive toxins. An example is given in the study focused on the simultaneous detection of ricin and abrin DNA by qPCR. Ricin and abrin DNA can be distinguished using differently labeled probes and fluorescence signals for ricin and abrin (Alx532) without crosstalk [204]. Other studies for the parallel detection of RIP plant and other protein toxins have been published [66,85,183,205,206].

Abrin from *Abrus precatorius*. Abrin is a protein toxin obtained from the seeds of *Abrus precatorius* (jequirity bean), which is similar in structure and properties to ricin. The A- and B-chains have molecular masses of approximately 35.5 kDa and 30 kDa, respectively. Multiple isoforms of these proteins have been isolated, and it is now generally accepted that three isoforms are routinely isolated; the relative molecular weights for the entire molecules were determined as 64 kDa for abrin I and 63 kDa for abrin II and III [207]. Isoforms can occur due to post-translational modifications of proteins or individual peptide chains being encoded by multigene families. Variations within the A- and B-chains, as determined by cDNA sequencing [208] and amino acid sequencing [209], are indicative of multiple-gene control. Abrus agglutinins are also extracted from *Abrus precatorius*. These are tetramers of approximately 130 kDa. The agglutinins have high amino-acid-sequence similarity to abrin [210], which has significance for detection methods, in particular assays for utilizing polyclonal antibodies against abrin protein, which show cross-reactivity to agglutinins. The presence of agglutinins is not a factor for toxicity, as they are considered to have minimal toxicological significance [211].

• Detection of abrin

Analytical methodologies directed at determining abrin are relatively new. Direct testing of abrin protein has been performed by ELISAs [212,213,214], immunochromatographic strips [215,216,217], multiplex detection immunoassay [206] and electrochemiluminescence assays [213]. Indirect means of testing for abrin include real-time PCR to test for the genes encoding the protein [204] and determination of the small molecule abrine by LC-MS [183,218].

Viscumin from *Viscum album*. The poisonous properties of mistletoe (*Viscum album*) have been known since ancient times. The toxic lectin viscumin isolated from *Viscum album* [219] is a cytotoxic 61 kDa large protein that binds to the galactose residues of cell surface glycoproteins and may be internalized by endocytosis. Viscumin strongly inhibits protein synthesis by inactivating the 60S ribosomal subunit. The structure of this protein is very similar to other RIPs mentioned above (abrin, ricin). Methods for the simultaneous detection of viscumin toxin with other toxins were published. One example is the quantitative immunoassay of biotoxins on hydrogel-based protein microchips, where three-dimensional gel-based microchips with immobilized proteins were used for quantitative immunoassay of a series of plant (ricin and viscumin) and bacterial (staphylococcal enterotoxin B, tetanus and diphtheria toxins, and lethal factor of anthrax) toxins [220]. The chips were incubated with solutions containing one of the toxins and developed with a mixture of Cy3-labeled antibodies against all six toxins. Bright fluorescent signals were observed in gel elements containing immobilized antibodies against the applied toxin. The main factor that determined the sensitivity of parallel analysis was the selection of antibody pairs. In this study, both aspects of a good detection method were demonstrated—high sensitivity in the sandwich analysis of the corresponding toxins and no cross-reaction with other toxins and antibodies. The detection limit for viscumin calculated in parallel analysis using a mixture of the six labeled antibodies was 2 ng/mL, similar to single-antibody tests. Another study for quantitative determination of lectin using the enzyme-linked lectin assay in mistletoe preparation was published with a detection limit of 5 ng/mL [221]. Summary of methods relevant to characterization of selected plant protein toxins is shown in Table 7. 

### 3.5. Additional Underestimated Protein Toxins

Conotoxins are group of neurotoxic peptides isolated from the venom of the marine cone snail of the genus *Conus*. Conotoxins are peptides consisting of 10 to 30 amino acid residues and typically have one or more disulfide bonds. These patterns of disulfide bridges help to define the number of structural classes of conotoxins, of which μ-conotoxins, ω-conotoxins, and α-conotoxins constitute the major classes. They are soluble in water and acidic acetonitrile and have a variety of mechanisms of actions, most of which have not yet been determined [222]. However, it appears that many of these peptides modulate the activity of ion channels, particularly by blocking potassium and sodium channels in neurons [223]. In recent decades, conotoxins have been the subject of pharmacological interest. Research is being conducted to determine their potential therapeutic value in chronic pain control, Parkinson’s disease, and neuromuscular disorders [224]. Conotoxins appear on selected Agents and Toxins Lists from the CDC [87]. At first, the discovery of each new conotoxin required biochemical purification from venom. Unusual post-translationally modified amino acids were identified in the first group of conotoxins characterized. As more cDNA clones encoding conotoxins were clarified, a molecular cloning/PCR approach for identifying novel conotoxins became possible. The first venom peptide characterized only on the basis of the predicted sequence from a cDNA clone was ω-conotoxin MVIIC. However, one problem with this approach is that if the conotoxin is post-translationally modified, knowledge of the encoding DNA sequence does not necessarily permit an accurate prediction of which amino acids are modified [225].

The method developed for purified α-conotoxin GI in phosphate buffer was published [226]. In this method, a biologically active fluorescein derivative of *Conus geographus* α-conotoxin (FGI) was used in solution-phase-binding assays with two purified mAbs to detect the toxin in laboratory samples. For competitive ligand-displacement spin-column assays, FGI was premixed with various dilutions of unlabeled ligands and then incubated with the two mAbs (5A1 and 8D2) at room temperature. Competitive displacement assays showed that both mAbs specifically bound α-conotoxin GI with high avidity. Cross-reactivity with α-conotoxins M1 and S1 was not observed for either mAb in a direct ELISA. Another method [227] developed for *Conus anemone* venom (α-Conotoxins AnIA, AnIB, and AnIC) in buffer was published. An LC/MS analysis with diagnostic screening for the detection of peptides with posttranslational modifications revealed the presence of novel sulfated peptides within the R-conotoxin molecular mass range in *Conus anemone* crude venom. Three sulfated α-conotoxins (AnIA, AnIB and AnIC) were identified and are within the molecular mass range of other α-conotoxins (i.e., 1400–2200 Da).

Bungarotoxins are a group of closely related neurotoxic proteins of the three-finger toxin superfamily found in the venom of kraits, including *Bungarus multicinctus*. The α-bungarotoxin inhibits the binding of acetylcholine to nicotinic acetylcholine receptors; β- and γ-bungarotoxins act pre-synaptically, causing excessive acetylcholine release and subsequent depletion. Both α and β forms have been characterized, and the α form is similar to the long or Type II neurotoxins from other elapid venoms [228].

The identification of bungarotoxins is tightly connected with the study of venomics [229,230]. There have been several reports on the diagnosis of snake envenomation, among which ELISA appears to be the most practical and commonly used. A literature review of the various immunoassays developed for the detection of toxins and venoms indicated that the test must not only be specific and sensitive but also rapid, easy to handle and able to withstand wide variation in the environment and field [231]. In reference [232], a highly sensitive, specific, and simple avidin–biotin optical immunoassay (AB-OIA) for the detection of β-BuTx in whole blood, plasma, urine, and tissue homogenates was developed. The assay can detect toxin in whole blood without manipulation, and the entire assay can be performed at room temperature without sophisticated equipment. Affinity purified rabbit IgG anti-β-BuTx antibody was immobilized on an optically active silicon surface (SILIAS™ wafer). The test sample was incubated, and the antigen–antibody reaction was monitored by the addition of a biotinylated monoclonal antibody specific to the toxin, avidin–horseradish peroxidase (HRP) and tetramethylbenzidine substrate. The assay detected β-BuTx levels as low as 16 pg/mL in sample buffer and 100 pg/mL in whole blood or plasma. The AB-OIA was also used to quantitate the postmortem level of β-BuTx in various organs such as brain, liver, and kidney, as well as the tissue at the site of injection. An analytical method based on biosensors was used to detect β-BuTx [233]. In this report, an ISFET-based immunosensor for the detection/quantitation of β-BuTx using a murine monoclonal antibody (mAb15) against β-BuTx and urease conjugated rabbit antibodies specific to the toxin was developed. Antibody immobilization protocols were optimized using SEM and chip ELISA. The ISFET immunosensor detected toxin levels of approximately 15.6 ng/mL.

## 4. Conclusions and Future Perspectives

Based on previous texts, it is clear that a variety of methods are available for each of the reviewed toxins based on different detection principles (e.g., immunochemical assays, MS-based methods, functional assays, and chromatographic methods). In general, to improve the state of detection for a protein toxins, the following is required: (I) Upgrade the preanalytical phase, sample preparation procedures (decrease unfavorable matrix effects, avoid false negative, positive results); (II) and achieve the highest selectivity, robustness, and specificity possible and (III) acquire the most suitable reagents (e.g., purified protein standards, convenient antibodies) to develop the methods. Immunological assays that can detect an antigenic property of a protein are sensitive but inclined to cross-reactions and false-positive results. Molecular biology techniques such as PCR give only indirect evidence of the presence of a certain protein as it reacts to the encoding nucleic acid. Activity tests show a certain type of toxin reaction but not the chemical identity of the toxins. MS-based techniques give direct proof of the molecular structure by measuring the molecular mass, the amino acid sequence, and posttranslational modifications. In addition, modern bioinformatics and proteomics MS approaches, which were developed for the detection and identification of protein-based toxins in different kinds of matrices, have been successfully demonstrated in biodefense applications. However, none of the mentioned methods alone provided complete pictures of the protein-based sample. A combination of techniques for unambiguous identification and tests to measure the biological activity of a particular toxin is the best way to obtain the most information for complex samples.

## Figures and Tables

**Table 1:** Summary of methods relevant to the detection of SEs.

Target Protein	Method of Detection	References
SEs	Kitten emesis test	[53]
	Reversed passive latex agglutination kit	[70,71,72,73]
	Enzyme-linked immunosorbent assay	[58,59]
SEA	Double-antibody solid-phase enzyme immunoassay	[56]
	Enzyme-linked immunosorbent assay	[60]
SEB	Direct skin test	[54]
	Single-gel diffusion test	[74]
	Latex agglutination test	[75]
	Latex agglutination inhibition test	[76]
	Enzyme-linked immunosorbent assay	[57,62]
	Fluorescence-based immunoassay	[63]
	Immunoreactor-based competitive fluoroimmunoassay	[65]
	ELISA-Lab-on-a-chip	[67]
	Surface plasmon resonance	[68]
	Electrochemical immunoassay using enzyme-nanosilica-doped carbon nanotubes for signal amplification	[69]
SEA, SEB	Double-gel diffusion assay	[77]
	Avidin-biotin ELISA	[61]
SEC	Fluoroimmunoassay based on functionalized fluorescent core-shell nanoparticle labels	[64]
Staphylococcal enterotoxin genes	Polymerase chain reaction	[12]

**Table 2:** Mass spectrometry methods for detection of SEs in various matrices.

Toxin	Matrix	Detection (QqQ)	Standards (PSAQ)	LOD	LOQ *	Reference
SEB	Apple juice	(QTOF)	-	60 ng/mL	N/A	[81]
SEA	Chinese dessert (Coco-pearls)	+	+	N/A	N/A	[42]
SEB	Chicken meat	(QIT)	-	N/A	N/A	[82]
SEA	Milk	+	+	SEA: 2.5 ng/g	Milk: 2.5 ppb	[83]
SEB	Shrimp			SEB: 10 ng/g	Shrimp: 5 ppm	
SEA	Milk	+	+	SEA: 4 ng/g	N/A	[84]
SEB				SEB: 8 ng/g		
SEA	Soup	+	+	SEA: 78 ng/mL	N/A	[85]
SEB				SEB: 141 ng/mL		
SED				SED: 48 ng/mL		

Abbreviation: QIT: Quadrupole ion trap. QTOF: Quadrupole Time-of-Flight. N/A: not available. PSAQ Protein standard absolute quantification (isotopically labeled standard). * LOQ (limit of quantification) determined by applying FDA guidelines for bioanalytical method validation, i.e., accuracy between 80% and 120%, precision 20% and signal-to-noise ratio >5. When accuracy was <80% or >120% or precision was >20%, LLOQ (lower LOQ) was determined based on a signal-to-noise ratio >10. (+): detected, (-): not detected by QqQ or PSAQ.

**Table 3:** Summary of methods relevant to detection of *B. anthracis*.

Target of Method	Method of Detection	Reference
Gene	RT-PCR-F	[91]
Spores	Light transmission	[92]
	Fluorescence	[93]
	MS	[94]
	SERS	[95]
	Bead-based sandwich immunoassay	[96]
	FRET-flow cytometry	[97]
	RT-PCR-F	[98]
	ELISA flow cytometry	[99]
	Flow cytometry	[100]
	ECL	[101]
	Immunoradiometric assay	[102]
Protective antigen	Fluorescence	[103]
	ENIA	[103]
	AM	[104]
	AFM	[105]
	Antibody microarray	[106]
	ELISA	[107]
DNA	RT-PCR-F	[108]
	PCR	[109]
	Fluorescence	[110]
Lethal factor	MS	[111]
	Antibody microarray	[106]

Abbreviations: amperometry (AM); atomic force microscopy (AFM); enzyme linked immunosorbent assay (ELISA); electrochemiluminescence (ECL); europium nanoparticle-based immunoassay (ENIA); fluorescence resonance energy transfer (FRET); real-time polymerase chain reaction and fluorescence (RT-PCR-F); surface enhanced Raman scattering (SERS).

**Table 4:** Summary of methods relevant to characterization of BoNT.

Target of Method	Technique	Reference
Botulinum toxin activity	Mouse lethality assay	[119,120]
	Rat compound muscle action potentials test	[123]
Spores	Polymerase chain reaction	[124]
Genes	Polymerase chain reaction	[125,126,127,128]
BoNT	Radioimmunoassay	[129,130]
	Passive hemagglutination	[131,132]
	Gel diffusion assay	[133]
	Enzyme-linked immune sorbent assay	[136,137,138,139,140,141,142,143,144,145]
	Fluorescence resonance energy transfer technology	[146]
	In vitro fluorimetric assay	[147]
	Endopeptidase assay	[149]
	Endopeptidase-MS assay	[150,151,152]
	Mass spectrometry	[116,153,154,155]

**Table 5:** Diversity of *C. perfringens* toxinotypes and associated diseases.

Toxinotype	Major Toxins				Minor Toxins				Associated Disease	
	α	β	ε	ι	CPE	λ	θ	δ	Humans	Animals
A	++	−	−	−	+	−	+	−	Gangrene, GI diseases	Diarrhea (foals, pig) NE in fowl
B	+	+	+	−	−	+	−	+	NE	Dysentery in newborn lambs Hemorrhagic enteritis in neonatal calves and foals Enterotoxemia in sheep
C	+	+	−	−	+	−	−	+		NE in piglets Enterotoxemia in sheep
D	+	−	+	−	+	+	−	−		Enterotoxemia in lambs, sheep, calves and goats
E	+	−	−	+	+	+	−	−		Enterotoxemia in calves

Abbreviation: GI: gastrointestinal, NE: necrotic enteritis. –: no production of toxin, +: production of toxins, ++: strong production of toxins.

**Table 6:** Summarization of methods relevant to characterization of *Clostridium perfringens* and its toxins.

Target of Method	Technique	Reference
*C. perfringens* strains	ELISA	[165]
	MS	[170,177]
Gene	PCR	[166,170,172,173,174]
Toxins	MS	[167,168,181]
	EIA	[171,175,176]
	ELISA	[178,179]
	PCR	[180]
*Clostridium* toxin activity	Cytotoxicity assay	[182]

**Table 7:** Summary of methods relevant to characterization of selected plant protein toxins.

Target of Method	Technique	Reference
RIPs	MS	[183]
	Multiplex fluorescent magnetic suspension assay	[186]
	ELISA	[212]
	Hydrogen-based protein microchips	[220]
Biological activity of RIP toxins	Microtiter-based assay (luciferase luminescence test)	[184]
Ricin	MS	[85,185,193,195,200,201,202,203]
	ELISA	[187,188,198]
	Mouse model	[192]
	Luminescent assay	[194]
	Chemiluminescence-based microarray immunoassay	[66]
Ricinine	MS	[199]
Genes (abrin)	PCR	[204]
Abrin	ELISA	[213,214]
	Immunochromatographic assay	[215,216]
	MS	[218]
Viscumin	Enzyme-linked lectin assay	[221]
